# Epidemiology of Traumatic Spinal Fractures at Ibra Hospital, Oman: A Retrospective Study With Regional Comparisons

**DOI:** 10.7759/cureus.103672

**Published:** 2026-02-15

**Authors:** Moosa A Alwardi, Deep Parkash, Mohammed Abdulhameed, Khalid Alnairi, Abhijit Nair

**Affiliations:** 1 Radiology, Ibra Hospital, Ministry of Health, Ibra, OMN; 2 General Surgery, Ibra Hospital, Ministry of Health, Ibra, OMN; 3 Epidemiology and Public Health, Ibra Hospital, Ministry of Health, Ibra, OMN; 4 Anesthesiology, Ibra Hospital, Ministry of Health, Ibra, OMN

**Keywords:** epidemiology, expatriates, motor vehicle collisions (or mvcs), oman, rta-road traffic accidents, spinal fractures, trauma, traumatic spinal fractures

## Abstract

Background: Traumatic spinal fractures represent a significant public health burden, associated with high morbidity, mortality, and socioeconomic costs, particularly in regions with limited epidemiological data, such as the Middle East.

Objective: This article aims to describe the prevalence, demographic and injury characteristics, management, outcomes, and associated risk factors of traumatic spinal fractures among patients admitted to Ibra Hospital, Oman, and to compare key patterns with regional studies in the Gulf Cooperation Council (GCC) and Middle East and North Africa (MENA) region.

Methods: This retrospective review examined trauma records at Ibra Hospital from January 1, 2019, to December 31, 2023. Data were extracted on demographics, injury mechanisms, fracture location and type, neurological status, management, and outcomes. Descriptive statistics and chi-square or Fisher’s exact tests were used to assess associations (p < 0.05).

Results: Of 723 suspected cases, spinal fractures were confirmed in 133 (18.4%). The cohort was predominantly male (110 (82.7%)) with high expatriate representation (88 (66.2%)). Lumbar fractures predominated (56 (42.1%)), with motor vehicle collisions (MVCs) as the leading mechanism (78 (58.6%)), associated with expatriate status (p = 0.001). Expatriates showed higher occupational risks (p < 0.001). Neurological deficits were more common in cervical injuries (p < 0.001).

Conclusion: Localized data underscore the need for targeted road safety measures, workplace protections for expatriates, and enhanced trauma care in Oman to reduce the burden of spinal fractures.

## Introduction

Traumatic spinal fractures constitute a major public health issue, are associated with high rates of morbidity and mortality, and impose a significant socioeconomic burden [1]. This challenge is evident at Ibra Hospital in Oman, where such injuries form a substantial part of the trauma caseload. Globally, the annual incidence is estimated at 10.5 cases per 100,000 population, resulting in approximately 768,473 new cases worldwide [1,2]. These fractures often lead to neurological deficits and are further complicated by factors such as osteoporosis and advanced age, which impair functional outcomes and diminish quality of life [1]. Nearly half of all cases require surgical intervention, underscoring their profound social and economic impact [2]. Effective prevention relies on localized epidemiological data to inform policy, yet such information remains scarce for the Middle East, a region with distinct demographic and socioeconomic characteristics [3]. Existing regional studies report an incidence of traumatic spinal injuries ranging from 5.2 to 23.2 per 100,000 in the Eastern Mediterranean and Middle East and North Africa (MENA) regions, with mechanisms varying by economic context: road traffic accidents dominate in higher-income areas, while falls are more common in lower-income settings [3,4].

This study addresses this gap by examining the epidemiology of traumatic spinal fractures among trauma patients at Ibra Hospital in Oman’s North Sharqiyah Governorate. It aims to characterize the epidemiology of traumatic spinal fractures at Ibra Hospital, Oman, to provide evidence-based benchmarks for national prevention and clinical management strategies. Specific objectives were to determine the prevalence of confirmed traumatic spinal fractures among suspected trauma cases and to describe patient demographics and injury mechanisms; to examine fracture characteristics (location, type, neurological involvement) and their associations with key factors (e.g., mechanism, nationality); to describe management approaches, predefined hospital outcomes (length of stay, in-hospital mortality, neurological deficit presence, surgical requirement), and to identify risk factors in this population; and to compare these findings with available regional (Gulf Cooperation Council (GCC)/MENA) and selected global data on traumatic spinal fractures.

## Materials and methods

This retrospective observational study was conducted at Ibra Hospital, a secondary care facility in North Sharqiyah Governorate, Oman, serving a mixed population of locals and expatriates. The study involved a retrospective review of existing medical records of trauma patients. It was approved by the Ministry of Health Research and Studies Committee (Proposal ID: MoH/CSR/24/29218), with a waiver of informed consent due to its retrospective nature and use of de-identified data. The study was conducted in accordance with the ethical principles of the Declaration of Helsinki.

All trauma patients admitted to the emergency department or trauma ward from January 1, 2019, to December 31, 2023, were screened via the hospital’s electronic medical records (EMR) system (Cerner Millennium) and radiology picture archiving and communication system (PACS). Inclusion criteria were age ≥18 years, traumatic mechanism of injury, and clinical suspicion of spinal injury prompting diagnostic imaging (plain radiographs, computed tomography (CT), and/or magnetic resonance imaging (MRI) per institutional trauma protocol). Exclusion criteria included nontraumatic etiology (e.g., osteoporotic compression fractures without trauma history, pathological fractures), isolated soft-tissue injuries without bony involvement, and incomplete records (defined as missing essential data such as demographics, mechanism, or confirmatory imaging report).

A traumatic spinal fracture was defined as any bony vertebral injury (vertebral body, posterior elements, or processes) resulting from a traumatic mechanism, confirmed radiologically by at least one modality (plain radiographs for initial screening, CT preferred for bony detail, MRI for soft tissue or ligamentous assessment when indicated). Confirmation required a radiology report or clinical documentation of a fracture. Dislocations without fracture were excluded unless associated with bony injury.

Data extraction was performed independently by two trained reviewers (clinical researchers familiar with trauma documentation) using a standardized, prepiloted Excel template with predefined fields and drop-down options to minimize variability. Discrepancies were resolved through discussion and consensus. In cases of unresolved disagreement, a senior author (trauma specialist) adjudicated. To ensure quality, 20% of cases were randomly selected for double extraction and review. Missing data were minimal (<5% for primary variables) and were handled via complete-case analysis for inferential statistics. No imputation was performed.

Key variables and operational definitions were as follows. Demographics included age (continuous, calculated as years at the date of admission), sex (binary: male/female as per registration), and nationality (categorical: Omani vs. expatriate, defined as non-Omani nationality per hospital registration records, reflecting foreign citizenship or residency status typical for migrant workers in Oman). Injury mechanism was categorized as motor vehicle collision (MVC; including driver, passenger, or pedestrian involvement), fall from height (≥1 meter or documented as such), occupational or other (work-related injuries not classified as falls, e.g., heavy lifting, machinery accidents), or assault or violence (intentional interpersonal trauma). Fracture characteristics included spinal level (cervical: C1-C7; thoracic: T1-T12; lumbar: L1-L5; multilevel: involvement of >1 region; other: sacral or coccygeal) and fracture type or stability assessed using the Thoracolumbar Injury Classification and Severity (TLICS) score (calculated by the treating orthopedic or neurosurgical team based on morphology, posterior ligamentous complex integrity, and neurological status; score ≥5 typically indicated surgical consideration). Neurological status was documented using the American Spinal Injury Association (ASIA) Impairment Scale (A-E) from the admission neurological examination by neurosurgery or orthopedics. "Neurological deficit" was operationally defined as ASIA grades A-D (complete or incomplete motor or sensory impairment below the level of injury), with no deficit defined as ASIA E (normal). Management was conservative (nonoperative: cervical collar or thoracolumbar brace, analgesia, bed rest or mobilization, physical therapy) or surgical (any operative intervention including decompression, instrumentation, or fusion). Outcome measures (prespecified and measured during index hospital admission) included primary outcomes of hospital length of stay (continuous: days from admission to discharge or in-hospital death, reported as median (interquartile range)) and in-hospital mortality (binary: death during index admission from any cause), and secondary outcomes of presence of neurological deficit (at admission) and requirement for surgery (binary: yes or no during admission). No long-term outcomes (e.g., neurological recovery, functional status at discharge, complications beyond mortality, ICU admission, discharge disposition) were available in the records and thus were not analyzed.

Statistical analysis used descriptive statistics: frequencies and percentages for categorical variables; means ± standard deviation (SD) for normally distributed continuous variables; medians and interquartile ranges (IQR) for skewed data. Inferential statistics included chi-square tests (or Fisher’s exact test for cells with expected counts <5) to assess associations (e.g., mechanism vs. nationality, fracture location vs. mechanism, neurological deficit vs. level). The Mann-Whitney U test was used for nonparametric comparison of hospital stay between groups (e.g., surgical vs. conservative). Significance was set at p < 0.05 (two-tailed). No correction for multiple comparisons was applied due to the exploratory nature of the associations. All analyses were performed using IBM SPSS Statistics for Windows, Version 27 (Released 2020; IBM Corp., Armonk, New York).

Findings were contextualized through narrative comparison with published regional (GCC/MENA) and selected global epidemiological data on traumatic spinal fractures.

## Results

Demographics and injury mechanisms (Objective 1)

Of the 723 suspected spinal injury cases reviewed, fractures were confirmed by imaging in 133 patients, yielding a prevalence of 18.4% among suspected cases. The cohort was predominantly male (110 (82.7%)) and included a high proportion of expatriates (88 (66.2%)). The mean age was 35.4 ± 12.5 years. Motor vehicle collisions (MVCs) were the leading mechanism of injury (78 (58.6%)), followed by falls from height (32 (24.1%)), occupational or other injuries (15 (11.3%)), and assault or violence (8 (6.0%)). MVCs showed a strong association with nationality (χ² = 12.4, p = 0.001), occurring more frequently among expatriates (68% vs. 40% in Omanis), and with sex (χ² = 8.7, p = 0.003). Occupational injuries were almost exclusive to expatriates (p < 0.001, Fisher’s exact test) (Table 1).

**Table 1:** Demographic Characteristics and Injury Mechanisms (N=133) Values are presented as n (%) or Mean ± SD. *p < 0.05 is considered statistically significant. MVC: motor vehicle collision.

Characteristic	Values	Statistical notes
Age (years)	35.4 ± 12.5	-
Sex: male	110 (82.7%)	p<0.001* vs. general trauma population
Nationality: expatriate	88 (66.2%)	p=0.012* vs. local demographics
Mechanism: MVC	78 (58.6%)	χ²=12.4, p=0.001* (nationality); χ²=8.7, p=0.003* (sex)
Fall from height	32 (24.1%)	-
Occupational/other	15 (11.3%)	p<0.001* (Fisher’s exact, expatriates)
Assault/violence	8 (6.0%)	-

To improve accessibility for policymakers, future supplementary materials could include infographics visualizing key associations such as Mechanism of Injury by Nationality (emphasizing expatriate predominance in MVCs and occupational injuries) and Fracture Location vs. Neurological Deficit (highlighting cervical predominance in deficits).

Fracture characteristics and neurological status (Objective 2)

Lumbar fractures were the most common (56 (42.1%)), followed by thoracic (38 (28.6%)), cervical (25 (18.8%)), and multilevel or other (14 (10.5%)). Fracture location was associated with mechanism (χ² = 15.2, p = 0.002). Neurological deficits occurred in 18 patients (13.5%), with a significantly higher rate in cervical injuries (48% vs. 5% in lumbar, χ² = 18.9, p < 0.001).

Management and outcomes (Objective 3)

Management was conservative in 95 patients (71.4%), while 38 (28.6%) required surgery, which was strongly linked to the presence of neurological deficits (χ² = 22.1, p < 0.001). The median hospital stay was 12 days (IQR 7-21), with longer stays in surgical cases (p = 0.008, Mann-Whitney U test). In-hospital mortality was 4 (3.0%), associated with cervical or multilevel injuries (p = 0.04, Fisher’s exact test) (Table 2).

**Table 2:** Fracture Characteristics and Outcomes (N=133) Values are presented as n (%). *p < 0.05 considered statistically significant.

Characteristic	n (%)	Statistical notes
Location: lumbar	56 (42.1%)	χ²=15.2, p=0.002* (mechanism)
Thoracic	38 (28.6%)	-
Cervical	25 (18.8%)	-
Multilevel/other	14 (10.5%)	-
Neurological deficit	18 (13.5%)	χ²=18.9, p<0.001* (higher in cervical)
Management: conservative	95 (71.4%)	-
Surgical	38 (28.6%)	χ²=22.1, p<0.001* (linked to deficit)
Hospital stay, median (IQR)	12 (7-21) days	p=0.008* (longer in surgical)
In-hospital mortality	4 (3.0%)	p=0.04* (Fisher’s exact, cervical/multilevel)

## Discussion

This study achieved its stated objectives by providing the first detailed description of traumatic spinal fractures at a secondary care center in Oman, revealing a confirmation rate of 18.4% among suspected cases, with thoracolumbar predominance and an MVC-driven etiology featuring significant expatriate overrepresentation (Objectives 1-3). These patterns align with global trends of thoracolumbar dominance and MVC or fall mechanisms [1,2], but they highlight Oman-specific occupational and transport-related risks for expatriates, consistent with broader GCC observations [3,4]. The strong association between MVCs and expatriate status (p = 0.001) likely reflects labor migration patterns, reliance on road transport, and potential disparities in vehicle safety awareness or conditions, as evidenced by regional data showing that non-nationals face higher occupational and fall risks [3-5].

In comparison with regional studies (Objective 4), recent GBD analyses indicate rising burdens in North Africa and the Middle East due to urbanization, population growth, and falls or MVCs, with vertebral fracture incidence increasing modestly in some subregions and persistent high absolute burdens in middle-SDI countries [6,7]. Road traffic accidents account for 56.5-90.8% of traumatic spinal cord injuries in Saudi Arabia, with young males and thoracic or cervical levels commonly affected, mirroring our findings on MVC predominance and neurological risks in cervical cases [8,9]. A Kuwaiti level-2 trauma center study reported spinal fractures in 28.7% of traumatic cases, with road traffic accidents at 54.5% and lumbar predominance, closely paralleling our 18.4% confirmation and 58.6% MVC rate [3], particularly in younger adults, where MVCs remain dominant [10]. A nationwide study in Iran also highlights trauma-related spinal fractures with varying regional mechanisms [11]. Neurological deficits were predominantly cervical-linked in our cohort (p < 0.001), aligning with Saudi findings of higher deficit and disability risks in cervical/thoracic injuries [8,12]. Similar single-center patterns have been reported in other Saudi centers [13]. Conservative management prevailed (71.4%), with surgery reserved for deficits (p < 0.001), aligning with resource-appropriate approaches in secondary centers. Low in-hospital mortality (3.0%) suggests effective acute care, although long-term outcomes remain unassessed.

Strengths lie in providing the first detailed Oman-specific benchmarks with explicit definitions and prespecified outcomes, filling regional gaps [14].

The high proportion of expatriates (88/133, 66.2%), substantially exceeding their estimated 33-35% share of the North Sharqiyah Governorate population, underscores marked vulnerability in this group. MVCs were strongly associated with nationality (68% among expatriates vs. 40% among Omanis; p = 0.001), and occupational or other injuries were almost exclusively among expatriates (p < 0.001). Although detailed job titles or commute specifics were not routinely documented, the "Occupational or Other" category (11.3% overall) likely reflects high-risk sectors prevalent among migrant workers in Oman, such as construction, logistics, heavy industry, and manual labor, where falls from height, machinery accidents, and long-distance commutes in employer-provided or shared transportation are common. These patterns mirror GCC-wide evidence of elevated road and workplace risks among non-nationals due to socioeconomic factors, variable vehicle safety standards, limited safety training, and reliance on public or company transport [5]. Targeted interventions should include expatriate-focused road safety education, mandatory employer vehicle safety audits, fall prevention programs in construction, and improved occupational health surveillance to reduce these disparities.

Conservative management predominated (71.4%), with surgery (28.6%) reserved for cases with neurological deficits (p < 0.001). In Ibra Hospital’s secondary care setting, this reflects pragmatic triage: stable patients receive bracing, analgesia, and early mobilization, while unstable or neurologically compromised cases (e.g., TLICS ≥5 or ASIA A-C) are referred to tertiary centers (e.g., Khoula Hospital in Muscat) for advanced neurosurgical intervention. Challenges include limited on-site spine specialists, variable referral transport times for rural patients, and reliance on initial CT or plain radiographs before MRI availability. These factors contribute to a conservative preference for borderline cases to avoid unnecessary transfers. Standardized national guidelines should emphasize early TLICS or ASIA assessment, clear referral thresholds, and teleradiology for timely decision-making, providing a blueprint for other secondary centers in Oman.

The low in-hospital mortality (3.0%), mainly linked to cervical or multilevel injuries (p = 0.04), suggests effective acute care, likely supported by prompt emergency department resuscitation, rapid imaging per protocol, and stabilization or referral. While time-to-treatment (e.g., door-to-CT or door-to-surgery intervals) was not systematically recorded in this retrospective review, institutional protocols generally aim for imaging within hours of arrival, consistent with Oman’s trauma system focus on early intervention. Prospective collection of such metrics would strengthen the evaluation of secondary care efficiency.

A limitation of this hospital-based design is the inability to calculate true population-based incidence rates. Ibra Hospital serves North Sharqiyah Governorate (estimated catchment population approximately 350,000-400,000, including approximately 33-35% expatriates per recent national statistics). Using 133 confirmed cases over 5 years yields a crude estimated rate of approximately 7-8 per 100,000 annually, lower than some MENA estimates (5.2-23.2 per 100,000), but likely underestimated due to selection bias and unreported minor cases. Multicenter registry linkage would enable accurate incidence estimation.

Other limitations include the single-center retrospective design, potential selection bias from record completeness, lack of long-term follow-up or population-level incidence data, and absence of information on additional outcomes (e.g., complications, ICU admission, discharge disposition, functional recovery).

To facilitate future multicenter, prospective studies and support national trauma care guidelines, we propose a standardized minimum data set for traumatic spinal fractures in Oman, including demographics (age, sex, nationality); mechanism details (with commute or occupational subcategories); fracture classification (TLICS score); neurological status (admission or discharge ASIA grade); management (conservative vs. surgical, referral details); and outcomes (LOS, in-hospital mortality, discharge disposition). Uniform adoption would enable pooled analyses, trend monitoring, and evidence-based policy development.

These findings advocate for enhanced road safety campaigns (e.g., seatbelt enforcement, speed controls), targeted workplace safety for expatriates (e.g., fall prevention in construction), and standardized trauma protocols. Future multicenter studies in Oman and the GCC could identify modifiable predictors, evaluate interventions, and track trends amid economic and demographic shifts [7,15,16].

## Conclusions

Spinal fractures represent a significant burden at Ibra Hospital, primarily driven by MVCs with notable expatriate occupational risks. Targeted prevention through road safety measures and workplace protections, alongside improved trauma care, is essential to mitigate this burden in Oman.
